# Novel characterization of MRAS mutation-associated Noonan syndrome: Mild adult-onset hypertrophic cardiomyopathy combined with infective endocarditis: A case report

**DOI:** 10.1097/MD.0000000000046340

**Published:** 2026-01-09

**Authors:** Xiaoli Mou, Yong Liu, Yarong Zhang, Xinhe Cheng, Junshuai Feng

**Affiliations:** a Gansu Provincial Hospital, Lanzhou, Gansu, China.

**Keywords:** case report, hypertrophic cardiomyopathy, infective endocarditis, MRAS gene mutation, Noonan syndrome, RASopathy

## Abstract

**Rationale:**

Noonan syndrome (NS) is a RASopathy most frequently associated with mutations in HRAS, NRAS, KRAS, and RRAS2. The contribution of MRAS variants to NS pathogenesis remains poorly characterized, and infective endocarditis (IE) is extremely rare in patients with NS.

**Patient concerns:**

A 22-year-old woman presented with typical dysmorphic features of NS, including short stature, broad forehead, hypertelorism, low-set posteriorly rotated ears, and a broad neck. She developed fever and progressive exertional dyspnea.

**Diagnoses:**

Echocardiography demonstrated obstructive hypertrophic cardiomyopathy with vegetation located in the left ventricular outflow tract. Blood cultures grew Streptococcus mutans. Whole-exome sequencing identified a heterozygous MRAS c.203C>T (p.Thr68Ile) mutation affecting a highly conserved residue among RASopathy-associated GTPases, supporting the diagnosis of MRAS-associated Noonan syndrome complicated by infective endocarditis.

**Interventions:**

Antibiotic therapy was escalated to intravenous gentamicin (60 mg daily) combined with ceftriaxone in accordance with the 2023 European Society of Cardiology guidelines for infective endocarditis.

**Outcomes:**

After one month of intravenous gentamicin and ceftriaxone therapy, fever resolved, and follow-up echocardiography showed disappearance of the vegetation. Residual cardiac abnormalities persisted, including marked left ventricular hypertrophy with left ventricular outflow tract obstruction, left atrial enlargement, moderate mitral regurgitation, patent foramen ovale with minor shunting, and impaired diastolic function. Exertional dyspnea remained despite resolution of the infection.

**Lessons:**

This case expands the genotypic spectrum of Noonan syndrome by supporting MRAS mutations as pathogenic drivers. It also identifies infective endocarditis as a previously unreported complication in MRAS-associated NS with outflow tract obstruction, highlighting the importance of careful cardiac surveillance in patients with RASopathies.

## 1. Introduction

Noonan syndrome (NS) is an autosomal dominant genetic disorder with an incidence rate of approximately 1 in 1000 to 2500 live births.^[1]^ NS is caused primarily by mutations in genes of the rat sarcoma (RAS) and mitogen-activated protein kinase pathways.^[2]^ Mutations in genes such as PTPN11, SOS1, RAF1, KRAS, RIT1, NRAS, RRAS, CBL, SOS2, and LZTR1 account for most identified NS cases.^[2]^ Although muscle RAS oncogene homolog (MRAS) gene mutations have recently been reported to be associated with NS, more clinical evidence is needed to fully characterize this association.^[2]^ Significant variability in disease phenotype and severity is characteristic of NS. Typical features include distinctive facial dysmorphology, skeletal abnormalities, short stature, developmental delay, learning difficulties, and congenital heart disease.^[3–5]^ Nearly 80% to 90% of NS patients have concomitant cardiovascular diseases, including pulmonary valve stenosis (50–60%), hypertrophic cardiomyopathy (HCM) (20%), atrial septal defect (10%), aortic coarctation (10%), and patent ductus arteriosus.^[6,7]^ However, NS patients presenting with infective endocarditis (IE) are rarely reported.

Herein, we present a rare case of an NS patient with IE associated with an MRAS gene mutation. This case provides valuable insights for future studies on the genotypic–phenotypic correlations of MRAS in NS.

## 2. Case presentation

### 2.1. Chief complaints

**Figure 1.**
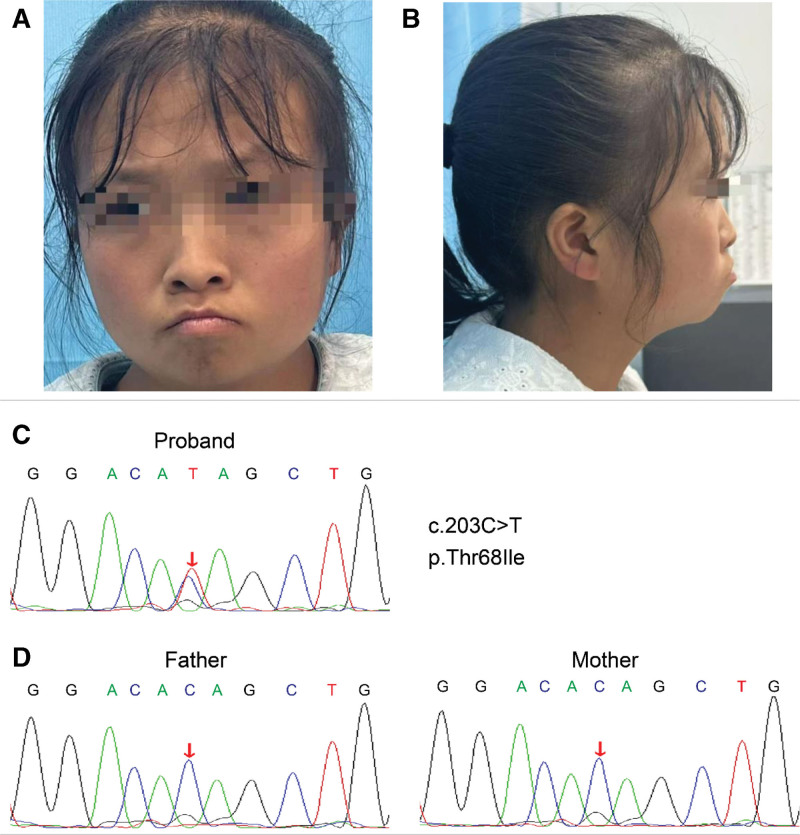
Facial characteristics of the patient and validation of *MRAS* variants by sequencing. (A) Front view of the individual at age 22. (B) Side view of the individual at age 22. The views show the facial characteristics of Noonan syndrome. (C) The sequence of the patient. (D) The sequence of the patient’s parents. MRAS = muscle RAS oncogene homolog.

A 22-year-old female presented with a 3-month history of intermittent fever (peaking at 38.5 °C), chills, exertional dyspnea, and persistent low-grade fever despite oral amoxicillin therapy (Fig. 1A and B).

### 2.2. History of present illness

Following 3 months of progressive symptoms, including activity-induced chest tightness and unresponsive fever, outpatient chest computed tomography (March 25, 2024) revealed no pulmonary infection, prompting hospitalization for fever of unknown origin investigation.

### 2.3. History of past illness

The patient had lifelong short stature (150 cm/37 kg) and childhood learning difficulties, with no documented cardiac abnormalities or significant prior hospitalizations.

### 2.4. Personal and family history

With no family history of congenital heart disease.

### 2.5. Physical examination upon admission

Febrile (38 °C) with characteristic dysmorphic features (broad forehead, hypertelorism, low-set posteriorly rotated ears, broad neck) and a harsh systolic ejection murmur at the left sternal border (3rd–4th intercostal space), while respiratory, abdominal, and extremity exams were unremarkable.

### 2.6. Laboratory examinations

Notable findings included neutrophilia, elevated procalcitonin and B-type natriuretic peptide, and *Streptococcus mutans* bacteremia in 3 blood culture sets (Table 1), with unremarkable autoimmune/tumor/tuberculosis markers, urinalysis, and fecal tests. Whole-exome sequencing confirmed a de novo heterozygous MRAS mutation (c.203C>T, p.Thr68Ile) absent in both parents (Fig. 1C and D).

**Table 1:** Laboratory examinations.

Test items	Specific items	Measured values	Normal values
Complete blood count	White blood cell (*10^9^/L)	7.9	3.5–9.5
	Red blood cell (*10^12^/L)	5.85↑	3.5–5
	Neutrophil (%)	81.2↑	50–75
	Lymphocyte (%)	12.1↓	20–40
	Basophil (%)	0.4	0–1
	Eosinophilic (%)	0.1↓	0.5–0.5
	Platelet (*10^9^/L)	178	100–300
	Hemoglobin (g/L)	106↓	110–150
Myocardial marker	Brain natriuretic peptide (pg/mL)	7898↑	<125
	High-sensitivity troponin I (ng/mL)	0.043↑	<0.0262
Bacterial infection	IL-6 (pg/mL)	34.36↑	<7
	Procalcitonin (ng/mL)	0.327↑	<0.065

IL-6 = interleukin-6.

### 2.7. Imaging examinations

**Figure 2.**
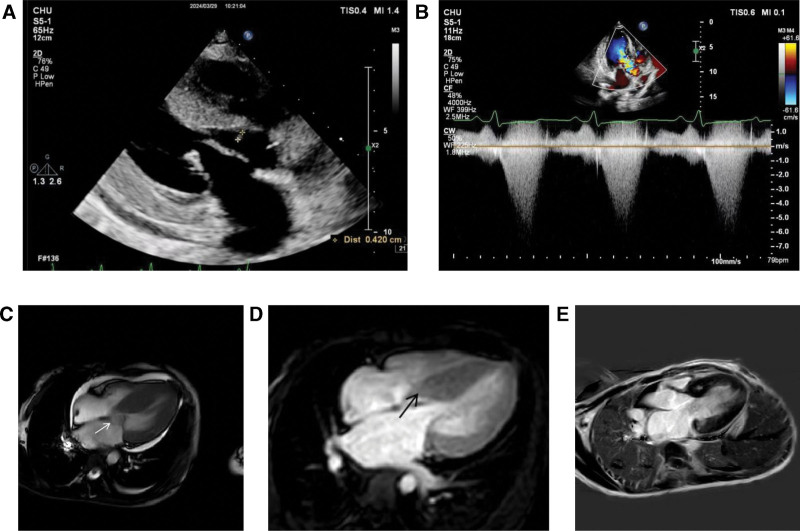
Echocardiogram and magnetic resonance imaging of the patient. (A) An echocardiogram shows vegetation (++) occupying the left ventricular outflow tract. (B) Echocardiogram shows left ventricular outflow tract obstruction. (C) MRI showed significant thickening of the ventricular septum and adjacent inferior wall and systolic mitral valve with SAM sign (white arrow). (D) First overperfusion showed a small lamellar area of reduced perfusion in the left ventricular septal wall (black arrow). (E) Delayed enhancement showed multiple focal intensifications in the interventricular septum. MRI = magnetic resonance imaging, SAM = systolic anterior motion.

Echocardiography revealed significant left ventricular wall hypertrophy with a slender filamentous vegetation attached to cardiac tissue 19 mm from the aortic valve, occupying the LVOT (Fig. 2A), Additionally, systolic anterior motion of the mitral valve leaflet caused dynamic LVOT obstruction (Fig. 2B). Associated findings included left atrial enlargement, pulmonary artery dilation, moderate mitral regurgitation, grade I diastolic dysfunction, mild pericardial effusion, and a patent foramen ovale with minor left-to-right shunting. Subsequently, the cardiac magnetic resonance imaging (MRI) was performed. The MRI confirmed asymmetric septal hypertrophy with systolic anterior motion of the mitral valve (Fig. 2C). Additional MRI findings included septal perfusion defects (Fig. 2D) and delayed gadolinium enhancement indicating myocardial fibrosis (Fig. 2E).

### 2.8. Diagnosis

The patient was definitively diagnosed with de novo heterozygous MRAS c.203C>T (p.Thr68Ile) mutation-confirmed Noonan syndrome, complicated by obstructive HCM with LVOT obstruction and IE meeting modified Duke criteria, evidenced by LVOT vegetations and *S mutans* bacteremia.

### 2.9. Treatment

Antibiotic therapy was escalated to intravenous gentamicin (60 mg daily) combined with ceftriaxone according to 2023 European Society of Cardiology guidelines, achieving fever resolution and normalized inflammatory markers within 5 days.

### 2.10. Outcome and follow up

Following 1 month of intravenous gentamicin/ceftriaxone therapy, the patient achieved complete resolution of fever with echocardiographic confirmation of vegetation disappearance. Persistent cardiac abnormalities included significant left ventricular hypertrophy, LVOT obstruction, left atrial enlargement, moderate mitral regurgitation, patent foramen ovale with minor shunting, and reduced diastolic function. Exertional dyspnea persisted despite resolution of infectious symptoms, necessitating ongoing cardiology follow-up for management of obstructive HCM.

## 3. Discussion

NS is an autosomal dominant disorder strongly associated with congenital heart defects, which contribute significantly to adverse prognoses and increased mortality. Approximately 80% to 90% of NS patients manifest cardiac abnormalities, among which pulmonary valve stenosis predominates (50–60% prevalence).^[8]^ Notably, IE in NS patients is exceptionally rare. To our knowledge, this represents the first documented case of IE in an NS patient harboring a pathogenic MRAS gene mutation.

The exceptional rarity of IE in NS, particularly left-sided IE arising from HOCM, demands careful consideration. This represents the first documented case of IE in an NS patient harboring a pathogenic MRAS gene mutation (c.203C>T, p.Thr68Ile). The confluence of several factors explains its occurrence here and its absence in prior reports: the patient’s mild, adult-onset obstructive HCM, a delayed manifestation specific to the MRAS p.Thr68Ile genotype contrasting with severe pediatric-onset disease associated with other MRAS variants and classic NS genes, allowed survival to adulthood with developing left ventricular outflow tract (LVOT) obstruction; this LVOT obstruction created high-velocity turbulent flow, causing endothelial injury at the obstruction site providing the essential nidus for vegetation formation (a risk distinct from right-sided lesions typical in NS); the absence of a prior NS or cardiac diagnosis likely meant no IE prophylaxis was administered for potential bacteremic events; and nonspecific initial symptoms in an undiagnosed syndromic adult may have lowered clinical suspicion, potentially delaying targeted investigation. This case establishes that pathogenic MRAS variants, particularly p.Thr68Ile, can cause mild, adult-onset HCM with significant LVOT obstruction, and that this specific phenotype confers substantial risk for left-sided IE due to endothelial trauma from turbulent flow. Therefore, in adults presenting with new HCM accompanied by obstruction and suggestive dysmorphic features, evaluation for underlying RASopathies such as Noonan syndrome should be considered. While standard cardiac care remains the priority for HCM, establishing a syndromic diagnosis allows for a comprehensive health strategy. This includes screening for associated noncardiac conditions, providing genetic counseling for the family, and tailoring monitoring protocols to include syndrome-specific risks, such as the heightened susceptibility to IE highlighted by this case. Such evaluation is advisable to enable holistic and preemptive patient management.

IE represents a rare complication of HCM, most frequently involving the mitral valve.^[9,10]^ The underlying pathophysiological mechanism involves high-velocity turbulent flow secondary to LVOT obstruction, causing endothelial injury that facilitates microbial colonization and vegetation formation.^[11]^ Consequently, LVOT obstruction and left atrial enlargement constitute established risk factors for IE development in HCM patients.^[12]^ Notably, IE occurrence in NS remains exceptionally rare, with only one prior documented case in the literature. This case represents the first report of IE in an NS patient harboring a pathogenic MRAS mutation (c.203C>T, p.Thr68Ile), underscoring the importance of vigilant cardiac monitoring in syndromic HCM patients, consideration of IE prophylaxis during high-risk procedures, and multidisciplinary management integrating cardiology, genetics, and infectious disease expertise for optimizing outcomes in this complex patient population.

Although NS represents a well-characterized monogenic disorder frequently diagnosed in clinical genetics practice, its complete molecular landscape remains incompletely defined. MRAS encodes a monomeric GTPase within the RAS superfamily that critically regulates mitogen-activated protein kinase and phosphoinositide 3-kinase–protein kinase B signaling cascades.^[13,14]^ To date, only 7 individuals with molecularly confirmed NS have been reported with pathogenic MRAS missense variants affecting codons p.Gly23, p.Thr68, and p.Gln71.^[2,15–18]^ We describe the 8th NS patient harboring the recurrent p.Thr68Ile variant. The affected residues (p.Gly23, p.Thr68, p.Gln71) exhibit high evolutionary conservation across RAS family GTPases associated with RASopathies, including HRAS, NRAS, KRAS, and RRAS2. This case reinforces p.Thr68 as a mutational hotspot, with p.Thr68Ile now representing 62.5% (5/8) of all known MRAS-associated NS cases. Our findings substantiate a robust genotype–phenotype association between MRAS pathogenic variants, particularly p.Thr68Ile, and the NS phenotype.

Among 7 reported NS patients with MRAS mutations, 6 pediatric cases manifested severe early-onset HCM characterized by aggressive progression, including neonatal cardiac arrest and premature cardiac death in 2 individuals.^[2,15–18]^ The inaugural adult case (p.Thr68Ile) was not documented until 2023, exhibiting only mild delayed left ventricular hypertrophy.

Affected pediatric patients uniformly manifested severe early-onset HCM characterized by aggressive progression, including neonatal cardiac arrest and premature cardiac death in 2 cases.^[2,15–18]^ The inaugural adult case (p.Thr68Ile) was not identified until 2023, demonstrating only mild delayed left ventricular hypertrophy. We report the second adult NS patient carrying this identical variant. This case fundamentally reshapes our understanding of MRAS-related cardiac pathology by demonstrating that HCM is neither obligate nor universally early-onset or severe; may first manifest in adulthood without antecedent cardiac pathology; and can coincide with novel complications like IE.

## 4. Conclusion

The evolving phenotypic spectrum of rare diseases presents significant diagnostic challenges. This case demonstrates that patients exhibiting characteristic NS facial features with cardiac involvement warrant genetic testing to confirm diagnosis. When such individuals develop unexplained sepsis-like manifestations, clinical suspicion for IE must be promptly raised, necessitating timely blood cultures, appropriate antibiotic therapy, echocardiography, and surgical intervention when major complications occur.

## Acknowledgments

The authors would like to thank the patient and her family for participating and approving our submission of this case report.

## Author contributions

**Investigation:** Xiaoli Mou, Yong Liu, Yarong Zhang, Xinhe Cheng.

**Writing – original draft:** Xiaoli Mou.

**Writing – review & editing:** Junshuai Feng.
